# Distinct Spiking Patterns of Excitatory and Inhibitory Neurons and LFP Oscillations in Prefrontal Cortex During Sensory Discrimination

**DOI:** 10.3389/fphys.2021.618307

**Published:** 2021-02-11

**Authors:** Hua-an Tseng, Xue Han

**Affiliations:** Biomedical Engineering Department, Boston University, Boston, MA, United States

**Keywords:** rodent prefrontal cortex, auditory discrimination, single unit activity, LFP oscillations, spike field coherence

## Abstract

Prefrontal cortex (PFC) are broadly linked to various aspects of behavior. During sensory discrimination, PFC neurons can encode a range of task related information, including the identity of sensory stimuli and related behavioral outcome. However, it remains largely unclear how different neuron subtypes and local field potential (LFP) oscillation features in the mouse PFC are modulated during sensory discrimination. To understand how excitatory and inhibitory PFC neurons are selectively engaged during sensory discrimination and how their activity relates to LFP oscillations, we used tetrode recordings to probe well-isolated individual neurons, and LFP oscillations, in mice performing a three-choice auditory discrimination task. We found that a majority of PFC neurons, 78% of the 711 recorded individual neurons, exhibited sensory discrimination related responses that are context and task dependent. Using spike waveforms, we classified these responsive neurons into putative excitatory neurons with broad waveforms or putative inhibitory neurons with narrow waveforms, and found that both neuron subtypes were transiently modulated, with individual neurons’ responses peaking throughout the entire duration of the trial. While the number of responsive excitatory neurons remain largely constant throughout the trial, an increasing fraction of inhibitory neurons were gradually recruited as the trial progressed. Further examination of the coherence between individual neurons and LFPs revealed that inhibitory neurons exhibit higher spike-field coherence with LFP oscillations than excitatory neurons during all aspects of the trial and across multiple frequency bands. Together, our results demonstrate that PFC excitatory neurons are continuously engaged during sensory discrimination, whereas PFC inhibitory neurons are increasingly recruited as the trial progresses and preferentially coordinated with LFP oscillations. These results demonstrate increasing involvement of inhibitory neurons in shaping the overall PFC dynamics toward the completion of the sensory discrimination task.

## Introduction

The prefrontal cortex (PFC) is known to be critically involved in decision making, and damage to the PFC leads to various cognitive deficits (Miller, 2000; Miller and Cohen, 2001; Dalley et al., 2004; Rossi et al., 2007; Gregoriou et al., 2014; Duan et al., 2015; Hanks and Summerfield, 2017; Gritton et al., 2020). PFC activity, both individual neuron’s spiking patterns and population local field potential (LFP) oscillation dynamics, have been correlated with many aspects of goal-orientated decision making process, such as attention, sensory processing, rule utilization, working memory, task progression tracking, and outcome anticipation. Recent studies using optogenetics to manipulate the activity of genetically defined cell types have showed that different PFC cell types are associated with distinct aspects of cognitive tasks (Hussar and Pasternak, 2009; Kvitsiani et al., 2013; Sparta et al., 2014; Murray et al., 2015). In general, excitatory neurons encode task components, whereas different subtypes of inhibitory neurons seem to be preferentially recruited during different stages of a task. Calcium imaging of PFC neurons in a go/no-go task further revealed that excitatory neurons exhibit heterogeneous responses, while inhibitory neurons tend to be more correlated within their subtypes (Pinto and Dan, 2015; Kamigaki and Dan, 2017), presumably due to gap junction coupling (Gibson et al., 1999). Parvalbumin-expressing (PV) neurons were shown to respond to various aspect of a task (Pinto and Dan, 2015; Lagler et al., 2016), especially to reward (Kvitsiani et al., 2013; Sparta et al., 2014), whereas somatostatin-expressing (SST) neurons tend to be more selective and respond primarily to sensory stimuli and motor activity (Kvitsiani et al., 2013; Pinto and Dan, 2015).

LFP oscillations in PFC have also been associated with a range of cognitive functions, and have been related to oscillations in other cortical and subcortical areas. In general, theta oscillations (∼5–10 Hz) are closely linked to working memory (Liebe et al., 2012), and are thought to coordinate long range connections between PFC and the hippocampus (Dejean et al., 2016). PFC Beta oscillations (∼15–30 Hz), largely associated with status-quo and rule application (Buschman et al., 2012), are often synchronized between PFC and other cortical areas. Higher frequency gamma oscillations (∼35–100 Hz) are found to be mainly local within the PFC, and are thought to be primarily involved in working memory (Lundqvist et al., 2016) and attention.

It has been suggested that LFP oscillations may organize neurons into functional ensembles (Watrous et al., 2015; Helfrich and Knight, 2016). For example, coherence between PFC spikes and sensory cortex LFPs was increased during covert attention, and spike-LFP coherence within the sensory cortex was found to be correlated with behavioral performance. Recent optogenetic studies showed that abnormal activity of inhibitory neurons can disrupt gamma oscillations in the PFC and lead to cognitive deficiency (Cho et al., 2015). While the PFCs in humans, monkeys, and rats are highly specialized, and extensively characterized during various cognitive tasks, the involvement of PFC in mice during cognitive behavior remains much less explored. A vast number of non-human primate studies have identified anatomically specified PFC subregions important for specific aspects of cognition. In contrast, the smaller mouse brain has fewer anatomically distinct cortical areas, and the mouse PFC is much less anatomically segregated and more functionally heterogenous. With the rapid progress of genetic tools developed for mice, mice are increasingly explored as a model organism for neural circuit analysis during behavior. However, it is largely unknown how mouse PFC neurons participate in cognitive behaviors. To investigate how mouse PFC neurons participate in sensory discrimination, we recorded both spikes and LFPs in the PFC, while mice were performing a 3-choice auditory discrimination task in an open field. With tetrode recordings, we identified well-isolated individual PFC neurons and distinguished putative excitatory neurons from putative inhibitory neurons using spike waveform features. We found that a large fraction of mouse PFC neurons and LFP oscillations were dynamically modulated during sensory discrimination, as observed in other animal models and humans. Inhibitory neurons were increasingly recruited toward trial completion, suggesting that the inhibitory neural population is particularly engaged as sensory discrimination progressed. In addition, we found that inhibitory neurons overall showed higher coherence with LFPs than excitatory neurons, highlighting the greater impact of inhibitory neurons over ensemble PFC population dynamics.

## Results

### A Majority of Individual PFC Neurons Exhibited Task Related Spiking Activities During a Three-Choice Sensory Discrimination Task

**FIGURE 1:**
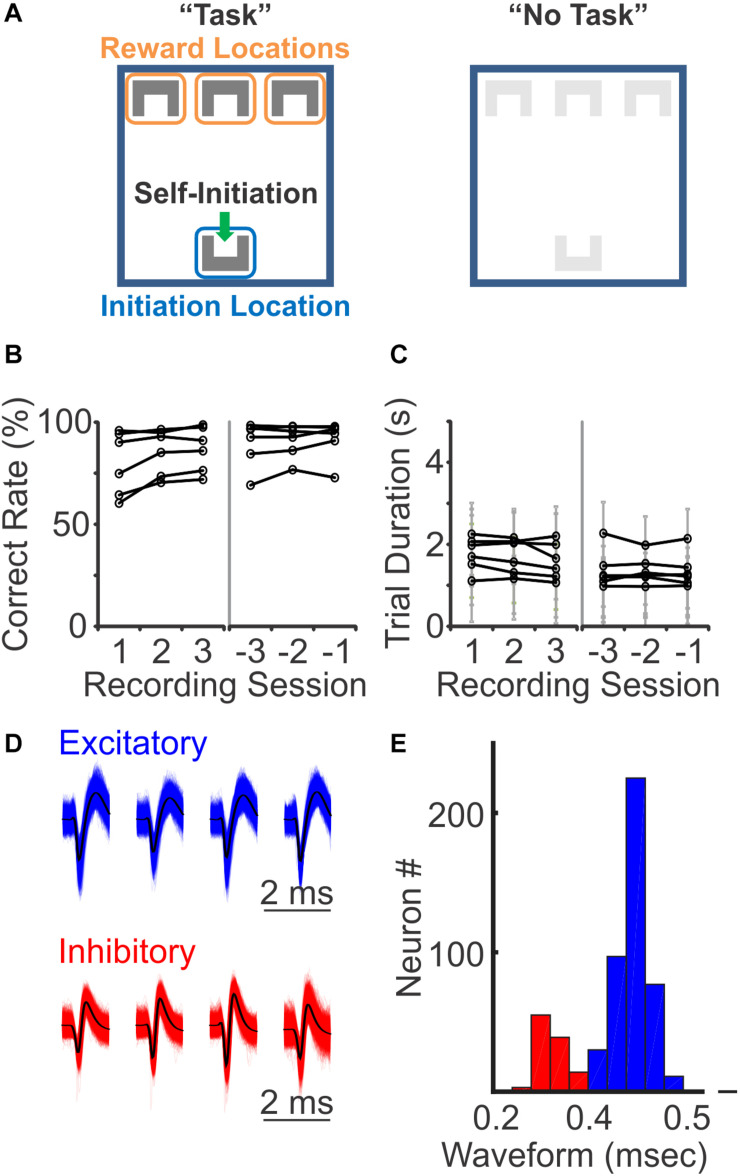
Three-choices auditory discrimination task and putative neuron classification. **(A)** During the auditory discrimination task, mice self-initiated each trial by triggering a motion detector at the “initiation” location (left, “Task”). Upon trial-start, one of the three auditory stimuli was initiated. On the other end of the arena, there were three reward locations, each paired with a specific auditory stimulus. Mice were given 5 s to reach one of the three reward locations. If mice reached the correctly paired reward location, a reward was provided. If mice failed to reach the correct reward location, they experienced a 5 s timeout period, with a bright light illuminating the arena. Auditory stimuli were played throughout the entire trial, until mice reached one of the reward locations or trigged the timeout period. During “No Task” blocks, both initiation and reward locations were covered with a different floor. The same auditory stimuli were present for 1.5–3 s, followed by a 1.5–3 s intertrial interval, while the mice were freely moving in the arena. **(B)** Behavioral performance during the first three recording sessions (left) and the last three recording sessions (right). All mice had correct rates above random chance of 33% (*N* = 6 mice). **(C)** The trial duration, the time from trial-initiation when mice self-initiate a trail at the initiation location until trail-completion when they reach the reward location, during the first three recording sessions (left) and the last three recording sessions (right). Each gray line represents mean ± standard deviation of an individual mouse. **(D)** Representative waveforms of excitatory and inhibitory neurons recorded with tetrodes. **(E)** Binomial distribution of the width of spike waveforms, with a clear separation at 0.4 ms between the two peaks, which was used as a threshold to identify putative excitatory (blue) and putative inhibitory (red) neurons.

To understand how distinct cell types are recruited during sensory discrimination, we designed a 3-choice auditory discrimination task (“Task”), which required freely moving mice to associate a specific auditory stimulus with a predefined “reward” location (Figure 1A). Briefly, mice self-initiated each trial by stepping into the “initiation” location to trigger one of the three auditory cues (10 kHz sine wave, 25 click/s, and 100 click/s), which was presented until trial completion. After trial initiation, mice were given 5 s to reach the “reward” location on the other end of the arena to receive a reward (correct trial). If mice reached the other two incorrect “reward” locations (incorrect trial) or failed to reach any “reward” location within 5 s (incomplete trial, excluded in this study), they were presented with a 5 s timeout, with bright light illuminating all “reward” locations. After training, all mice maintained a performance of >60% correct rate over the recording period (Figure 1B). Most mice were able to complete each trial within 3 s, with an average time of 1.31 ± 0.57 s across all completed trials in 6 mice (Figure 1C). This auditory discrimination task involves identification of auditory information, association of auditory stimuli and reward location, and locomotion to the rewarded location.

We performed 251 recording sessions in 6 mice, with tetrodes targeting PFC bilaterally, and identified 711 single neurons based on simultaneously recorded waveforms from four closely positioned tetrode wires. Among these 711 neurons, 552 (78%) showed significant changes in their firing rates during the task, when compared to their activity during the inter-trial interval (ITI) (*p* < 0.05, Wilcoxon rank sum test). To understand how different PFC neurons are selectively modulated during the sensory discrimination task, all subsequent analysis was performed on responsive neurons only. To explore the difference in excitatory and inhibitory neural responses, we sorted the recorded individual neurons based on their spike waveforms. Of the 552 task-relevant neurons, the width of their spike waveforms followed a bi-normal distribution, with one peak centered at 0.45 ms, consistent with the general observation of putative excitatory neurons, and another peak centered at 0.25 ms, consistent with the general observation of putative inhibitory neurons (Figures 1D,E; Cardin et al., 2007; Mitchell et al., 2007; Han et al., 2009; Lee et al., 2017). Thus, based on spike width, we identified 441 excitatory neurons (80%) and 111 inhibitory neurons (20%), using 0.4 ms as a threshold. The recorded excitatory neurons exhibited a lower average firing rate comparing to the inhibitory neurons (excitatory: 1.66 ± 1.84 Hz, inhibitory: 2.62 ± 2.68 Hz; *p* < 0.05, Wilcoxon Ranksum test), consistent with the corresponding characteristics of these two neuron populations.

**FIGURE 2:**
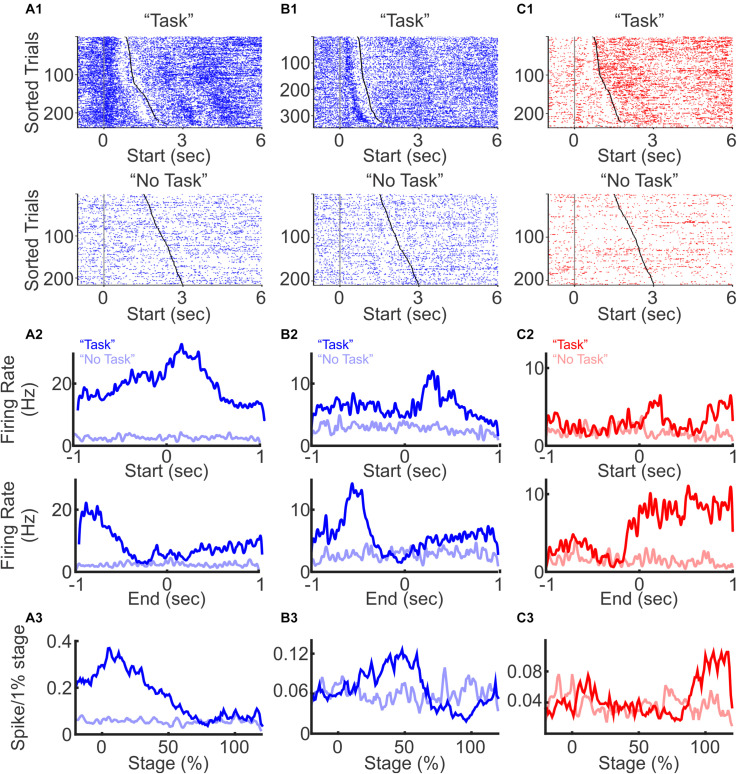
Excitatory and inhibitory neuron spiking during a three-choice auditory discrimination behavioral task. **(A)** A representative excitatory neuron with elevated firing rates at trial start. Raster plots of spike activities from all trials were aligned to the trial start during the auditory discrimination “Task” block (**A1** top) and during the “No Task” block (**A1** bottom). Trials were sorted by trial durations from the shortest to the longest. Each blue or red dot represents a spike, and the gray and black dots indicate trial start and trial end, respectively. The average firing rates of the same neuron across all trials during the auditory discrimination tasks (dark line, “Task”) and during “No Task” (light line), aligned to the trial start (**A2** top), and to the trial end (**A2** bottom). Normalized firing rates throughout the entire trial duration of the “Task” block (**A3** dark line) and of the “No Task” block (**A3** light line). **(B)** Similar to **(A)**, but from a representative excitatory neuron with elevated firing rate in the middle of the task. **(C)** Similar to **(A)**, but from a representative inhibitory neuron with elevated firing rate at the trial end.

We first examined the timing of PFC spiking relative to the stage of a trial. When aligned to trial start, some neurons showed an immediate increase in firing rates at trial start with firing rates gradually decaying toward the end of the trial (Figure 2A, with individual trials sorted by trial duration from shortest to longest for visualization), some exhibited an increase after trial start and primarily fired during the middle of the trial (Figure 2B), and some exhibited small increase at trial start followed by a sharp rise toward the end of the trial (Figure 2C). Given the variable duration for a mouse to complete each trial, to capture dynamic changes of PFC neurons during different stages of trial progression, we normalized PFC firing rate as percent activity change throughout the entire trial length, from trial initiation to trial completion. We found that individual neuron firing rates were dynamically modulated at different stages of the task. However, as a population, PFC neurons responses were present across all stages of the trial, suggesting that the PFC is engaged throughout the entire sensory discrimination task period (Figures 2A3,B3,C3).

To confirm that the observed PFC activity was indeed task related, we included a “No Task” block, where mice were placed in the same arena, but with a different floor that covered all the sensors at the “initiation” and “reward” locations. The same auditory stimuli were played, but mice cannot access the operant and reward components of the discrimination task during the “No Task” block. The “No Task” block contained 200–250 trials. During each trial, one of the same three auditory stimuli was randomly presented for a duration of 1.5–3 s, followed by a 1.5–3 s long ITI. Trial duration for the “No Task” condition was defined as the duration when auditory stimuli were present. To be able to compare the activity of the same neuron during the discrimination task vs. without the task, the “No Task” block was performed either before or after the “Task” block in the same recording session. Of the 422 task-modulated neurons that were also recorded during the “No Task” block, only 12 (3%) neurons showed significant changes in firing rate (Figure 2). Together, these results demonstrate that a large fraction of PFC neurons are selectively modulated during the sensory discrimination task, but not during the “No Task” condition with passive presentation of sound. In addition, although individual PFC neurons are transiently activated during different stages of the task, the overall PFC neural dynamics is engaged throughout the entire task period.

### Inhibitory Neurons Were Preferentially Modulated Toward Trial Completion

**FIGURE 3:**
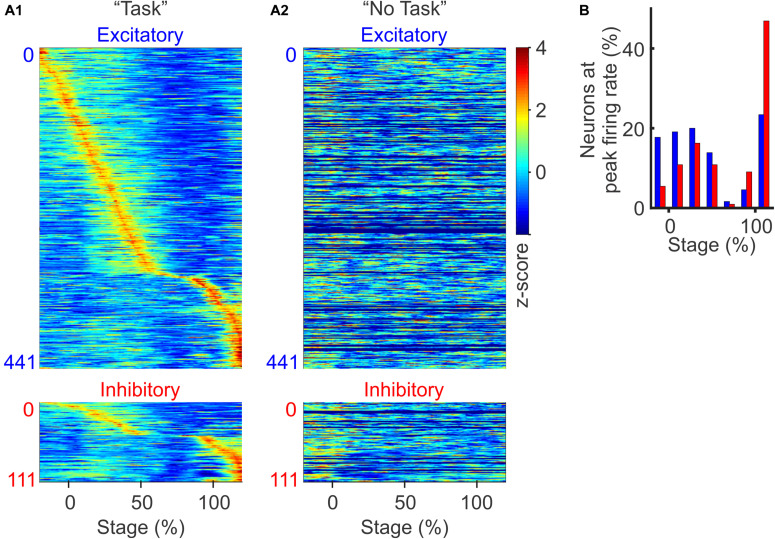
Context and stage-dependent modulation of spiking activity. **(A)** Normalized population firing rates during the discrimination task **(A1)** and during the “No Task” condition (**A2**, sorted in the same order as in **A1**. The 130 neurons recorded only in auditory discrimination task without corresponding “No Task” block were filled with dark blue). Neurons were grouped by type (excitatory and inhibitory) and sorted based on the timing of their peak firing rates. For each neuron, the firing rate was normalized by its *z*-score over the −20 to 120% stage. **(B)** Distribution of excitatory (blue bars) and inhibitory neurons (red bars), based on the timing of their peak firing rate during the task (*p* < 0.01, χ^2^-test).

To examine how excitatory and inhibitory neurons are differentially modulated during the discrimination task, we sorted neurons according to the relative timing of their peak firing rates. We found that both excitatory and inhibitory neurons were transiently modulated throughout the task from trial initiation to reward retrieval, with different neurons exhibiting peak firing rates at different phases of the task (Figure 3A1). Excitatory neurons tended to be active throughout the entire trial period, with two-thirds of neurons peaking in activity during the beginning 50% of the trial. In contrast, we found substantially more inhibitory neurons were recruited toward the end of the trial (Figure 3B; *p* < 0.01, χ^2^ test, between the distribution of excitatory and inhibitory neurons). PV positive inhibitory neurons are strongly activated when animals are approaching reward, receiving reward, and these neurons can encode trial outcome (Kvitsiani et al., 2013; Sparta et al., 2014). Our results are consistent with these previous findings. Not only did we find increased activation of inhibitory neurons toward discrimination task completion, but such increase in inhibitory activity was also accompanied with the suppression of local PFC excitatory neuron activity.

When we examined these same task responsive PFC neurons during the “No Task” block, we found that none of these cells were significantly modulated, confirming that PFC neurons exhibit task-specific modulation, rather than simply responding to bottom-up auditory stimuli alone (Figure 3A2). While PFC neurons are known to respond to auditory stimuli during passive conditions (Romanski and Goldman-Rakic, 2002; Miller et al., 2015), in our study, the three auditory stimuli at ∼70 dB delivered over an ambient environment of ∼60 dB were not sufficient to evoke significant passive responses in the PFC neurons.

We further compared excitatory vs. inhibitory neuron firing rates during correct vs. incorrect trials, and found that both neuron subtypes showed clear sequential activity during the correct trials (Supplementary Figure S1A1), but not during the incorrect trials (Supplementary Figure S1A2), confirming that both excitatory and inhibitory neurons contribute to encoding task outcomes. Together, these results demonstrate that both excitatory and inhibitory neurons in the PFC encode task stage specific information that correlates with behavioral outcome. The fact that an increasing fraction of inhibitory neurons are recruited toward trial completion, accompanied with suppressed excitatory neural activities, suggests that inhibitory neurons exhibit greater influence over PFC network dynamics toward the completion of the auditory discrimination task.

### PFC Neurons Encode Auditory Stimulus Identify, and Inhibitory Neuron Populations Exhibit Increasing Discrimination Ability Toward the Completion of the Trial

**FIGURE 4:**
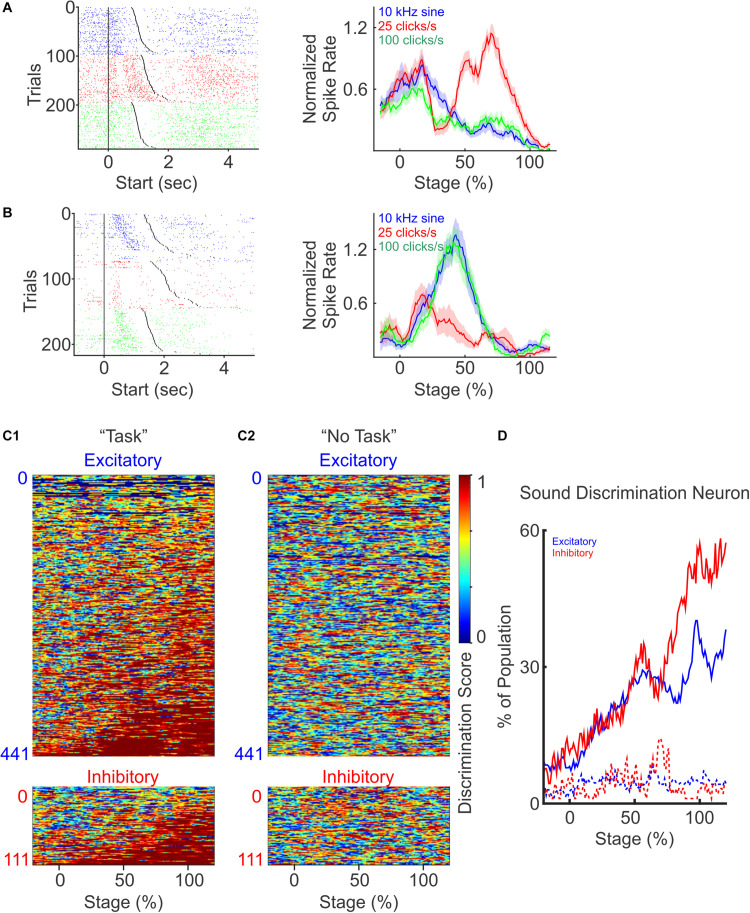
PFC neurons discriminate different auditory cues. **(A)** A representative neuron with increased response to the presentation of one auditory cue (25 click/s), but not to the other two (10 kHz sine wave and 100 click/s). Left: spike raster plot for trials presented with different auditory cues (blue: 10 kHz sine wave; red: 25 click/s; green: 100 click/s), and sorted by trial durations. Right: normalized firing rate during each auditory stimulus across trials. **(B)** Another example neuron responded to two auditory cues (10 kHz sine wave and 100 click/s), but not to the third (25 click/s). **(C)** Discriminatory ability of each neuron presented as discrimination scores, defined as one minus the *p*-values calculated with one-way ANOVA between the firing rates to the presentation of the three auditory cues at different trial stages. Neurons were grouped as excitatory (top) and inhibitory (bottom), and sorted by the total duration when they were discriminative. Discriminative responses of individual PFC neurons only occurred during the discrimination task **(C1)**, but not during “No Task” condition (**C2**, the neurons are sorted in the same order as **C1**). **(D)** The percentage of excitatory (blue) and inhibitory (red) neurons showing sound discrimination throughout trial stages.

While PFC neurons are broadly tuned to auditory stimuli, they are known to discriminate sensory stimuli and to categorize sensory inputs (Freedman et al., 2001; Miller and Cohen, 2001; Lee et al., 2009). To examine whether mouse PFC neurons are selectively modulated by different auditory cues, we compared their responses to the three cues used in the task (Figures 4A,B). We observed that PFC neurons exhibited highly heterogeneous responses to different auditory cues. Some PFC neurons responded to only one auditory cue but not to the other two (Figure 4A), whereas some responded to two cues but not to the third one. This demonstrates that individual PFC neurons can encode cue identity with highly heterogeneous response profiles, highlighting that PFC networks could utilize the heterogeneity of individual neurons to expand the coding capacity of a large variety of cues using a population coding strategy. Consistent with the observation that PFC spiking responses are dynamic during the task, tone specific responses were often restricted to certain stages of the task.

To quantify the auditory cue selectivity of each PFC neuron, we calculated the discrimination score, defined as 1 minus the *p*-value from One-way ANOVA test, between the firing rates upon the presentation of the three auditory cues at the different stages of the trial. A larger selectivity score indicates that the firing rates of a neuron exhibit greater difference in response to different auditory cues. We then plotted the selectivity scores of each neuron over the entire trial length, and calculated the fraction of neurons that exhibit significant discriminatory selectivity during the trial (Figures 4C1,D). Overall, we found that an increasing number of excitatory and inhibitory neurons become more sound discriminative as the trial progressed (Figure 4D). However, inhibitory neurons show an enhanced ability to distinguish tone identity later in the trial compared to the excitatory neurons, with ∼50% of the interneuron population being auditory stimulus selective by trial end (Figure 4D, excitatory: dark blue, inhibitory: dark red, χ^2^−test, *p* < 0.01). During the “No Task” condition, these same PFC neurons did not discriminate sound identity (Figure 4C2), and the percentage of modulated neurons stayed low and constant throughout the auditory stimuli presentation (Figure 4D, dot lines). Together, these results demonstrate that both excitatory and inhibitory PFC neurons’ spiking activity evolves to encode the identity of sensory stimuli when it was relevant to the sensory discrimination task, consistent with the idea that PFC activity plays a prominent role in facilitating the identification and utilization of sensory stimuli. We found that the proportion of inhibitory neurons that ultimately exhibits sensory discrimination was greater than the excitatory neuron population (Figure 4D), and this discrimination coincided with the period of peak activity of interneurons near the end of the trial period (Figure 3B), both of which suggest a greater impact of inhibitory neural population in facilitating sensory discrimination.

### Excitatory and Inhibitory PFC Neurons Are Differentially Coordinated With PFC LFP Oscillations at Different Frequency Bands

**FIGURE 5:**
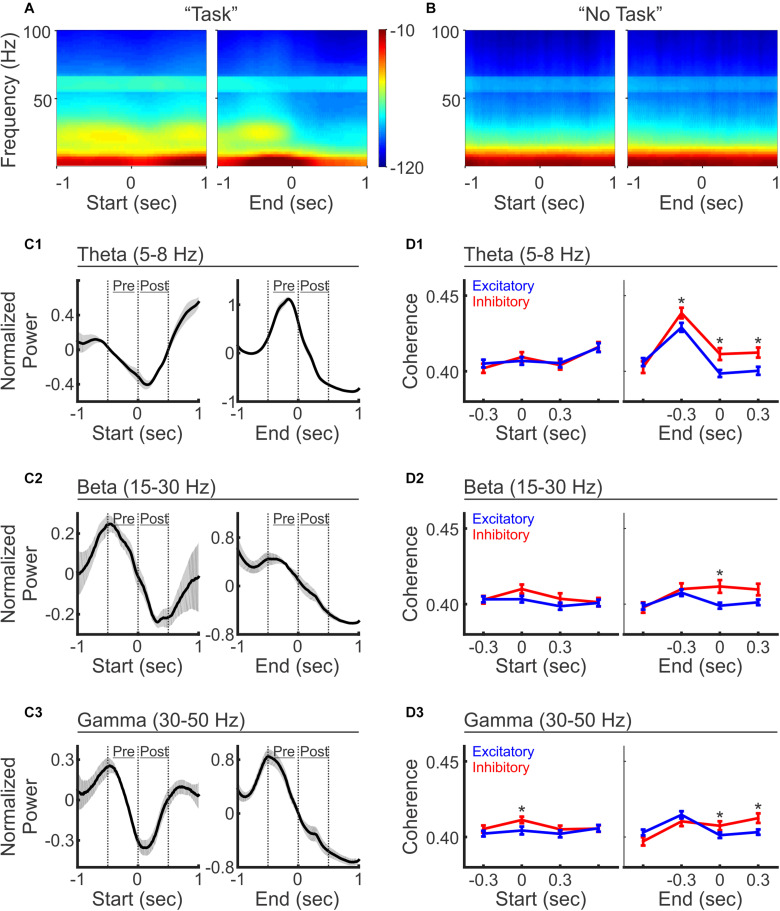
LFP oscillations and selective coherence with excitatory vs. inhibitory neurons during auditory discrimination. **(A)** Average LFP power spectrum during the auditory discrimination task from one example recording session, aligned at trial-start (left) and trial-end (right). **(B)** Average LFP power spectrum during “No Task” block from the same recording session as **(A)**. **(C)** Normalized LFP oscillation powers at theta (**C1**, 5–8 Hz), beta (**C2**, 15–30 Hz), and gamma frequencies (**C3**, 30–50 Hz), aligned at trial-start (left), and at trial end (right), during auditory discrimination (black line). LFP powers were normalized as *z*-scores to the 2 s window analyzed. Shaded areas indicate standard error (*N* = 5 animals). **(D)** Spike-field coherences of excitatory neurons (blue, mean ± standard error of mean) and inhibitory neurons (red, mean ± standard error of mean), aligned at trial-start (left), and at trial-end (right), during the discrimination task, at theta **(D1)**, beta **(D2)**, and gamma frequencies **(D3)**, aligned to trial start (left) or to trial end (right). (Excitatory = 331 neurons, inhibitory = 91 neurons, two tailed, unpaired, *t*-test, *p < 0.05).

PFC LFP oscillations have been broadly associated with many cognitive functions. We next examined LFP power across a variety of frequency ranges to examine whether mouse PFC LFPs are modulated during the auditory discrimination task. We observed that LFP power across all frequencies was transiently decreased upon trial initiation, and then slightly recovered in the middle of the trial, followed with a sharply decrease again by trial end (Figure 5A). To further quantify LFP oscillation changes during different phases of the trial progress, we compared the power at Theta (5–8 Hz), Beta (15–30 Hz), and Gamma (30–50 Hz) frequencies. Upon trial initiation, the reductions in frequency powers were significant for all frequency bands analyzed (Figure 5C; comparison between the averaged powers during the 500 ms time windows pre and post trial start: *N* = 5 mice, paired *t*-test, *p* < 0.05). At trial end, the decrease in frequency power was also broad brand across all frequencies analyzed (Figure 5C; comparison between the averaged powers during the 500 ms time windows pre and post trial end: *N* = 5 mice, paired *t*-test, *p* < 0.05). Conversely, during “No Task” block, LFP power analysis revealed a small but significant increase across all frequencies at trial start (*N* = 5 mice, paired *t*-test, *p* < 0.05), in contrast to the reduction in oscillation powers at this stage during the discrimination task block (Figure 5B, *N* = 5 mice, paired *t*-test, *p* < 0.05).

While we observed LFP power changes across multiple frequency bands, inhibitory neurons have been linked to oscillations at specific frequencies (Cho et al., 2015). To investigate how spiking patterns were temporally associated with particular LFP frequencies, we calculated spike-field coherence between individual neurons and the LFPs recorded from the adjacent electrode within the ipsilateral hemisphere (Figure 5D). Interestingly, while LFP power is reduced across all frequencies at trial start, the reductions differentially impacted the coherence of excitatory vs. inhibitory neurons. Inhibitory neurons exhibited significantly higher coherence only at gamma frequencies, but not at theta and beta frequencies (Figure 5D). This result is similar to the finding that inhibitory neurons are important for PFC gamma oscillations during rule shifting behavior (Cho et al., 2015), highlighting the general coupling of inhibitory neurons and PFC gamma oscillations during cognitive tasks. The fact that gamma frequency power is reduced at this stage, but inhibitory neurons were more coherent with gamma frequencies, highlights that the PFC inhibitory neurons show greater coupling to gamma oscillations at the initiation of the sensory discrimination task even though gamma power is relatively reduced.

As trial progresses toward completion, spike-field coherence at theta frequency increased in both neuron types, with inhibitory neurons showing a higher coherence than excitatory neurons, which sustained beyond trial completion (Figure 5D1). At beta and gamma frequencies, the divergence of spike-field coherence between excitatory and inhibitory neurons only occurred after the trial end, where inhibitory neurons again showed stronger coherence than excitatory neurons (Figures 5D2,D3). In summary, these results demonstrate that inhibitory neurons show stronger coherence with LFPs than excitatory neurons across multiple frequency bands during the behavioral task, suggesting a more coordinated inhibitory neuron network irrespective of the frequency band analyzed. It is possible that different inhibitory neuron subtypes are preferentially recruited at distinct timepoints within the task, which could account for the observation of the spike-field coupling with distinct frequency components as previously suggested (Gibson et al., 1999; Pinto and Dan, 2015; Kamigaki and Dan, 2017).

### PFC LFP Oscillation Changes Are Behavioral Outcome-Dependent

**FIGURE 6:**
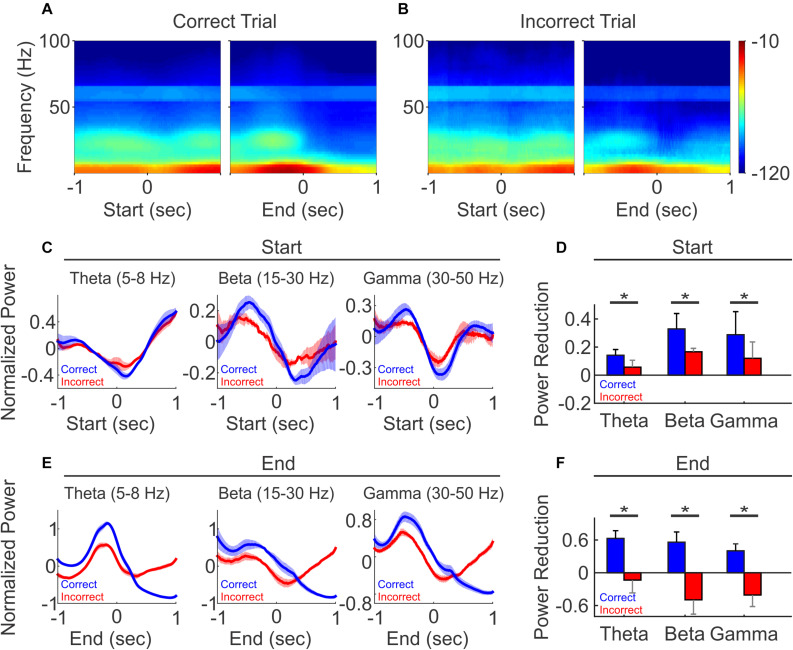
LFP oscillation power diverged between correct and incorrect trials. **(A)** Spectrogram of the correct trials from one representative recording session, aligned to trial-start (left) and to trial-end (right). **(B)** Spectrogram of the incorrect trials from the same recording session as **(A)**, aligned to trial-start (left) and to trial-end (right). **(C,E)** Normalized population LFP oscillation powers of correct (black line) and incorrect (gray line) trials, aligned to trial-start **(C)** and to trial end **(E)**. **(D,F)** The difference of LFP power, defined as the averaged *z*-scores of 500 ms windows before minus after at trial start **(D)**, at trial end **(F)**. (*N* = 5, paired *t*-test, *p < 0.05).

To understand the role of LFP oscillations in task performance, we investigated whether LFP oscillations were differently modulated by task outcomes. When aligned to trial start, LFP oscillation power showed similar trends during the correct and incorrect trials (Figures 6A left vs. 6B left). We then further quantified the dynamics of LFP power at trial start, by comparing the average power at theta, beta and gamma frequencies during the 500 ms time window before trial start vs. the 500 ms window after trial start. We found that while the power is reduced on both correct and incorrect trials, the reduction is greater on correct trials than incorrect trials, across all frequencies (Figures 6C,D, *N* = 5 mice, paired *t*-test, ^∗^*p* < 0.05).

Interestingly, at trial end, changes in oscillatory power diverged based on the trial outcome. On correct trials, oscillation powers continued to decrease and remained low over a prolonged period into the ITI, across all frequencies (Figure 6E, blue line). For the incorrect trials, oscillation powers first decreased, similar to that observed in the correct trials, but then rose across all frequency bands (Figure 6E, red line). As a result, the power changes at the trial end were bifurcated and significantly different, where the averaged powers showed reductions in correct trial, but exhibited an opposite relationship on incorrect trials (Figure 6F, *N* = 5 mice, paired *t*-test, ^∗^*p* < 0.05). Together, these results demonstrate that PFC LFP oscillation activity is linked to task performance, and the prolonged difference in LFP oscillations after each trial may serve as a feedback signal associated with reporting trial outcome.

## Discussion

Understanding the different dynamics of excitatory vs. inhibitory neuronal populations in PFC is of great interest in studying PFC involvement in cognitive functions. Here we designed a three-choice auditory discrimination task, and performed tetrode recordings from well-identified individually PFC neurons in task performing mice. We distinguished putative excitatory neurons and putative inhibitory neurons based on spike waveforms, and found that inhibitory neurons on average showed peak activity later in the trial compared to excitatory cells. For both neuron populations, encoding of auditory stimuli was prominent during the discrimination task but not when these stimuli were irrelevant. These results demonstrate that both excitatory neurons and inhibitory neurons were recruited in a context-dependent manner, across different stages of the auditory discrimination task. Not only did we detect an increasing fraction of inhibitory neurons activated as the trial progressed, but we found that inhibitory neurons as a population were more selective in auditory discrimination and preferentially associated with LFP oscillations. Together, these results demonstrate that inhibitory neuron populations play a prominent role in shaping overall PFC dynamics during sensory discrimination.

PFC neurons exhibit diverse response profiles during cognitive tasks, from task progression (Ma et al., 2014), to different aspects or stages of a task (Asaad et al., 1998). PFC neurons modulate their firing rates with varying temporal dynamics, when anticipating task-relevant sensory stimulation (Rodgers and DeWeese, 2014), responding to sensory stimuli presentation (Ninokura et al., 2004; Russ et al., 2008a; James et al., 2019), maintaining working memory (Rainer et al., 1998; Romo et al., 1999; Constantinidis et al., 2001; Kopec et al., 2015), predicting and/or anticipating reward (Matsumoto et al., 2003; Toda et al., 2012), or outcome (Amiez et al., 2006; Russ et al., 2008b; Hayden et al., 2011; Del Arco et al., 2017), which can linger into the inter-trial interval (Marcos et al., 2016). We here show that mouse PFC neurons exhibit transient increases at specific stages of a 3-choice discrimination task, suggesting that different PFC neurons can be sequentially recruited to process task-relevant information. The 3-choice auditory discrimination task allows us to examine more complex features of PFC encoding ability. We found that while some neurons increased their firing rate specifically to only one auditory cue, others could be modulated by a combination of cues. The ability for individual neurons to respond to multiple cue combinations could expand PFC population coding capacity, as each specific sensory information could be collectively represented from the combined selectivity of an ensemble of neurons.

In our experiment, mice were provided with different environmental contexts for the “Task” blocks, and the “No Task” blocks. Although mice were presented with the same auditory stimuli in both blocks, mice only had access to the operant and reward apparatus during the “Task” blocks, but not the “No Task” blocks. In our study, we found only 3% of PFC neurons were modulated during the “No task” blocks, in contrast to the 78% observed during discrimination task blocks, confirming that PFC neural activity is dependent on the context of the discrimination task. Such context-dependent modulation has been shown when animals were exposed to different environments, or were performing tasks requiring different rules. However, it is possible that PFC activity could represent the association rules between the auditory stimuli and the reward locations. These rules were context-dependent and were only utilized during the task. Alternatively, PFC could also be involved in motor planning to obtain the reward. For example, even though in the open-space square box area, mice could follow different traveling path from the start location to the reward location to complete each trial, mice may develop fixed movement patterns associated with specific auditory stimuli. Thus, it is difficult to assigned specific PFC neuron activity patterns to a particular task component, beyond the overall context of the task. In addition, the mice may exhibit different movement patterns during the “Task” block and during the “No Task” block, which may result in the difference in the observed PFC activity between the two conditions, since movement is an inherent component of our “Task” condition. The difference in context-dependent PFC activity observed here may also involve many neuromodulators, such as acetylcholine and dopamine that have been shown to mediate attentional states.

Over the years, many *in vivo* electrophysiology studies have classified neurons into putative excitatory and inhibitory groups based on extracellularly recorded spike waveforms, since morphological, genetic and molecular features informative for cell classification are not accessible by extracellular recording electrodes. Spike waveform based cell classification is broadly supported by evidence from *in vitro* brain slice recordings and *in vivo* intracellular recordings in animals, which have in general demonstrated that excitatory neurons, and a very small fraction of interneurons, have wide action potentials that can be recorded as broad spike waveforms extracellularly, whereas fast-spiking inhibitory neurons exhibit narrow action potentials leading to narrow spike waveforms recorded extracellularly (McCormick et al., 1985; Kawaguchi and Kubota, 1997; Henze et al., 2000; Gonzalez-Burgos et al., 2005; Mitchell et al., 2007; Ferrante et al., 2017; Keaveney et al., 2020). Using spike waveform-based classification method, many *in vivo* studies have revealed distinct physiological functions between putative excitatory vs. inhibitory neurons. However, waveforms alone remain an incomplete classification method. For example, cultured excitatory and inhibitory neurons could exhibit indistinguishable action potential width, likely due to the developmental features unique to cell cultures (Weir et al., 2014), in contrast to the results of many *in vitro* brain slice studies (McCormick et al., 1985), highlighting the importance of considering a wide range of features in cell classification. Recent *in vivo* recording studies also demonstrated that many somatostatin positive interneurons exhibited similar spike width as excitatory neurons (Ma et al., 2010; Weir et al., 2014; Li et al., 2015), even though some somatostatin positive interneurons have been shown to exhibit narrow action potentials (Yavorska and Wehr, 2016; Keaveney et al., 2020). It is possible that our classified putative excitatory neurons with broad waveforms include subpopulations of somatostatin positive or other interneuron subtypes, but these interneurons represent a very small fraction of the overall putative excitatory cell population (Rudy et al., 2011; Pfeffer et al., 2013). Thus, the waveform classification method deployed here should allow us to capture the major differences between the excitatory neuron populations and the inhibitory neuron populations.

Different types of PFC neurons have been shown to exhibit distinct task-related responses (Hussar and Pasternak, 2009; Hussar and Pasternak, 2012; Murray et al., 2015). Excitatory neurons tend to increase their firing rates with less variability upon sensory stimulation, whereas inhibitory neurons are more correlated with the later stage of a task, such as reward (Kvitsiani et al., 2013). Most recently, using optogenetics, Sparta et al. (2014) demonstrated that selective activation of PV neurons facilitated extinction learning that involves the dissociation of cue-reward relationships. We found that an increasing proportion of neurons, both excitatory and inhibitory, became discriminative to the identity of the auditory cues as trials progressed, suggesting gradual recruitment of PFC neurons during sensory discrimination consistent with the general idea that the PFC accumulates information during decision making (Freedman et al., 2001). Interestingly, we also found that inhibitory neurons showed higher spike-field coherence than excitatory neurons. Inhibitory neurons have been implicated in supporting LFP oscillations in the PFC (Lodge et al., 2009; Kim et al., 2016) and other brain areas (Cardin et al., 2009; Miller et al., 2015). Our observation that a larger fraction of inhibitory neurons exhibited cue selectivity, together with the finding that inhibitory neurons possessed a stronger spike-field coherence, suggest that inhibitory networks have increasing impact as discrimination progressed.

While we found that LFP power was altered similarly across multiple frequency bands, the coherence between LFP oscillation and spike activity was however neuron type specific. Inhibitory neurons showed stronger spike-field coherence than excitatory neurons at higher LFP oscillations frequencies (gamma) at task onset but at lower frequencies (theta) during the trial. The fact that inhibitory neurons exhibited a higher degree of coherence across all frequency bands is suggestive of inhibitory neural networks in supporting PFC oscillations, which may play a crucial role in organizing cell assemblies within the PFC in a context-dependent manner as postulated for the general functional significance of LFP oscillations.

LFP oscillations at specific frequencies have been related to different aspects of behavioral tasks and states, and LFP synchrony within the PFC and between the PFC and other brain areas has been observed in many tasks (Antzoulatos and Miller, 2016). In addition to single neuron responses in the PFC, we also observed wide spread changes in LFP power across all frequencies. Even though oscillations across multiple frequency band seem to have similar power dynamics during the task, a more detailed examination revealed that their coherences with the excitatory and inhibitory neurons differ, depending on the task stage and the frequency band analyzed. Moreover, the dynamics of LFP power were also associated with task performance. As LFPs capture the collective contribution from populations of nearby neurons, changes in LFP dynamics could be indicative of enhanced coherence amongst ensembles of PFC neurons crucial for different aspects of the task for successful task performance.

In general, our results showed that mice PFC encodes task-relevant information in a context dependent manner, similar to the PFC in other species (Hyman et al., 2012; Ma et al., 2016). Interestingly, comparing to excitatory neurons, inhibitory neurons exhibit higher coherence with LFP oscillations across all frequencies, highlighting their roles in shaping overall PFC population activity during the task, likely via direct connections to surrounding excitatory neurons within the PFC. The exact mechanisms by which inhibitory neurons modulate overall PFC network dynamics remain elusive and need further investigation. In our study, it is possible that as mice move toward a reward location, inhibitory neurons encoding the rewarded location can actively suppress excitatory neurons representing other non-rewarded location choices. In fact, we detected concomitant suppressions of excitatory neuron populations at the later stage of a trial, when inhibitory population responses increased, which provides some support, though indirect, for local interactions between excitatory and inhibitory neurons within the PFC. It is also possible that sound discriminatory inhibitory neurons could further increase the fraction of sound discriminatory excitatory neuron at trial end, by suppressing the firing rates of excitatory neurons to different auditory stimulus. Future exploration of these specific hypothesis using causal optogenetic analysis could reveal informative insights on the interaction dynamics between excitatory and inhibitory neurons within the PFC.

## Materials and Methods

### Three—Choice Auditory Discrimination Task

#### Behavior Arena

The behavior arena (12 × 12 inch) was constructed with black plastic walls and a mesh floor. The start location was located at one side of the arena (1 inch away from the wall), and three reward locations were at the opposite side (1 inch away from the wall) (Figure 1). Each location was equipped with an IR beam sensor and a white LED light on the floor. A speaker was located outside of the box, at the reward side, and delivered a 70 db auditory cue when the animal initiated the task. The room had a consistent background noise of approximately 60 db. LabVIEW (2012, National Instruments, Austin, TX) scripts were used to control the sensors and LED lights in the behavior arena via a NiDAQ board (NI USB-6259, National Instruments, Austin, TX) and recorded behavior events. For the “No Task” behavioral condition, a black plastic floor was placed above the IR beam sensors to prevent animal access to these apparatuses.

#### Behavioral Training

All animal procedures were approved by the Boston University Institutional Animal Care and Use Committee. Female C57BL/6 mice (Taconic, Hudson, NY), were water-restricted during behavioral testing, and were closely monitored to ensure that they maintained at least 85% of their pre-experiment body weight. Adult female mice (2–3 months old at the start of the experiments) were trained to perform the 3-choice auditory discrimination task in following steps:

Step 1: Obtain water from reward port. At this step, only the middle reward port was accessible to the animal. It delivered a water reward whenever an animal reached the reward location and triggered the IR beam sensor in front of the water port.
Step 2: Detection of the first auditory cue. A white LED at the start location was illuminated to indicate trial initiation. Mice learned through trial and error to initiate a trial by reaching the start location, which triggered the IR beam sensor and the first auditory cue was presented. Mice were allowed to reach the reward location to obtain the reward without any time limit.
Step 3: Detection of the second auditory cue. After the animal learned initiating the task and responding to the first cue, we made the reward location associated with the second cue accessible, while blocking the reward locations for the other two cues. At this step, when animals initiated a trial, only the second cue was presented, and animals were required to reach the corresponding location for reward with no time limit.
Step 4: Two-choice auditory discrimination task. At this step, the reward locations for both the first and the second cues were accessible. When animals initiated the trial, one of two auditory cues would be presented randomly, and mice were required to reach the corresponding reward location for reward with no time limit. When animals reached the incorrect reward location, a 5 s timeout occurred, indicated by white LED lights around the reward locations.
Step 5: Detection of the third auditory cue: After the animal learned the 2-choice discrimination task, we repeated Step 3 to introduce the third auditory cue. At this step, only the third reward location was accessible and the other two were blocked. When an animal initiated the trial, only the third auditory cue was played, and the animal was required to reach the third reward location to complete the trial and receive the reward with no time limit.
Step 6: Three-choice auditory discrimination task. All three reward ports were accessible. When animals initiated the trial, one of the three auditory cues was presented, and mice were required to reach the corresponding reward location to obtain reward. At this stage, a trial duration limit of 5 s was introduced. Failure of reaching the reward location within 5 s would cause a timeout, indicated by white LED lights around the reward locations. To obtain a balanced number of trials with each cue, auditory cues were presented in a group of three, and within each group, each auditory cue was presented once in a random order.

During training, each mouse was trained 20 min per day. Once well-trained, defined as performing over 60% correct rate per day over 3 consecutive training days and capable of completing a minimum of 100 trials per day, animals were provided free water access in their home cage for a week and then underwent tetrode implantation. After tetrode implantation and recovery from surgery, animals were briefly re-trained using the procedures described in Steps 2–6 until their performance reached 60%, and then recordings were performed.

### Electrophysiology

Custom tetrode devices (16 channels) were assembled in house, which contained four tetrode bundles, two bundles targeting each hemisphere. The four tetrode bundles were designed to target the PFC bilaterally (AP: +1.8, ML: +0.2; AP: +2.2; ML +0.2; AP: 2.2; ML: -0.2; AP 2.2, ML: +0.2). A tetrode bundle was made by twisting together 4 tetrode wires (Sandvik-Kanthal, Ahmerst, NY), and adjusting the impedance to 0.5–1 MOhm with gold plating (SIFCO 5355, SIFCO ASC, Independence, Ohio). Tetrodes were implanted with the center positioned at the midline (AP: 2, ML: 0), so that the tetrode bundles targeted the PFC bilaterally (AP: 2+/-0.2, ML: +/- 0.2). We advanced tetrode bundles gradually during the re-training period, so that the tip of the tetrode bundles reached the PFC at the recording stage (AP: 1.0–1.9). All recordings were performed in freely-moving mice. During recording, the tetrode device was connected to a commutator (ACO32, Tucker-Davis Technologies, Alachua, FL) to ensure free movement in the behavior arena. The raw wideband signals were acquired with a Plexon OmniPlex system at 40 kHz (Plexon Inc, Dallas, TX). The Plexon OmniPlex recorded local field potentials at 1 KHz sampling rate using a low pass filter at 200 Hz, and spike signals at 40 kHz sampling rate using a high pass filter at 300 Hz. Individual spike waveform snippets were obtained by amplitude-thresholding the continuous spike signals with a manually selected threshold value in the negative range during recording. The Plexon OmniPlex system also received time stamps from a NiDAQ board (National Instrument) to record timing of behavioral events. The recorded LFP, spike waveform, and behavior timestamps were then analyzed offline as described below.

Mice underwent one recording session a day. Each recording session constituted one “discrimination task” block and one “No Task” block in random orders. Animals could move freely in the behavioral arena during the entire recording session. The “discrimination task” block lasted for about 20 min, and animals were allowed to initiate the task as many times as they desired. In general, mice performed 100–200 trials within 20 min. During the “No Task” block, mice were placed in the same behavioral arena with a plastic floor positioned above all IR beam sensors and LEDs, so that mice had no access to any sensors or water ports. The same three auditory cues (∼70 db) used in the “discrimination task” were played in pseudo-random order for 200–250 trials. The durations of the auditory cues were randomized from 1.5 to 3 s with random 1.5–3 s inter-trial intervals.

### Data Analysis

Spikes were sorted with Offline Sorter (Plexon Inc, Dallas, TX) and then imported into Matlab (2014, MathWorks, Natick, MA) for further analysis. Spike width was defined as the duration between the valley and the peak of a spike waveform. Due to the difference in lengths of each trial, to calculate the firing rate throughout trial progression, we first normalized each trial based on its duration, so that trial start and trial end were aligned at 0 and 100% of trial progression, respectively. We then calculated the firing rate from −20 to +120% of trial progression using a 1% moving window, and smoothed the results by averaging each data point with its two adjacent data points. When presenting the population data, we further normalized the firing rate between −20 and 120% of trial progression by calculating the z-scores for each neuron with its own mean and standard deviation.

LFPs were imported into Matlab with the Matlab custom script provided by Plexon, and then analyzed with the Chronux toolbox^1^. The power spectrogram of each LFP trace was calculated with mtspecgramc function [moving window size: 500 ms, moving window step: 5ms, tapers: (Ninokura et al., 2004)] in Chronux. In a few occasions, animal movement caused artifacts in our tetrode recording, such as when the tetrode devices bumped the arena walls, which created large voltage deflections in our recordings. To eliminate the impact of such movement artifact in our analysis, we identified these artifact periods as having at least 5% maximum amplitude, either positive or negative, of the whole recording session. Trials that contained these artifact periods were excluded from all analysis involving LFPs.

The spectrogram examples were log-normalized [10^∗^log(power/max)], with the maximum power of the examined time window (2 s, centered at either trial start or end). To compare the powers at specific frequency bands across mice, the powers within the examined time window (2 s, centered at either trial start or end) of each trial were first normalized by converting to their *z*-scores and then averaged within a given frequency band to obtain the power for each trial. The power across all trials from the same animal were then averaged for each animal. In our analysis, we examined three frequency bands, and the ranges of each frequency bands were defined as: theta (5–8 Hz), beta (15–30 Hz), and gamma (30–50 Hz).

Spike-field coherence was calculated with cohgramcpt function [moving window size: 500 ms, moving window step: 5ms, tapers: (Ninokura et al., 2004)] in Chronux toolbox^1^. For each neuron, we calculated spike-field coherence (session coherence) between its spike activity and the ipsilateral LFP recorded at another electrode during the entire recording session. To obtain trial averaged coherence of each neuron at selected frequency bands at trial start, we first aligned the coherences at trial start and then averaged across trials. The trial averaged coherence values within the desired frequency band were then averaged across neurons, and binned with the 300 ms time window, centered at trial start. The population coherence was calculated by averaging the population coherence at selected frequency band and time point. To obtain the population coherence at selected frequency band at trial end, we aligned the session coherence to the trial end and performed the same procedure as described above.

### Statistical Testing

For spike rate modulations, we used one-way ANOVA to compare the firing rates at the same trial progression period for trials with different auditory cues. For LFP power spectrum comparisons, we used a paired *t*-test to compare the power during the 500 ms before and after either trial start or trial end. For spike-field coherence, we used a non-paired *t*-test to compare the coherence at the same trial progression period between the “discrimination task” blocks and the “No Task” blocks. All analyses were performed in Matlab. Details for each test are presented throughout the section “Results.”

## Data Availability Statement

The raw data supporting the conclusions of this article will be made available by the authors, without undue reservation, to any qualified researcher.

## Ethics Statement

The animal study was reviewed and approved by the Boston University Institutional Animal Care and Use and Biosafety Committees.

## Author Contributions

HT and XH designed all experiments and wrote the manuscript. HT performed experiments and data analysis. XH supervised the project. Both authors contributed to the article and approved the submitted version.

## Conflict of Interest

The authors declare that the research was conducted in the absence of any commercial or financial relationships that could be construed as a potential conflict of interest.

## Footnotes

1 chronux.org

## Supplementary Material

Supplementary Figure S1 PFC spiking activity during correct vs. incorrect trials. (Related to Figure 2). Normalized population firing rates of excitatory (blue) and inhibitory (red) neurons during correct trials **(A1)**, and incorrect trials **(A2)**.
