# Supplementary figures and images for: Distinct Spiking Patterns of Excitatory and Inhibitory Neurons and LFP Oscillations in Prefrontal Cortex During Sensory Discrimination
Source: Front Physiol. 2021 Feb 11;12:618307. doi: 10.3389/fphys.2021.618307 (PMC7928411; doi:10.3389/fphys.2021.618307)

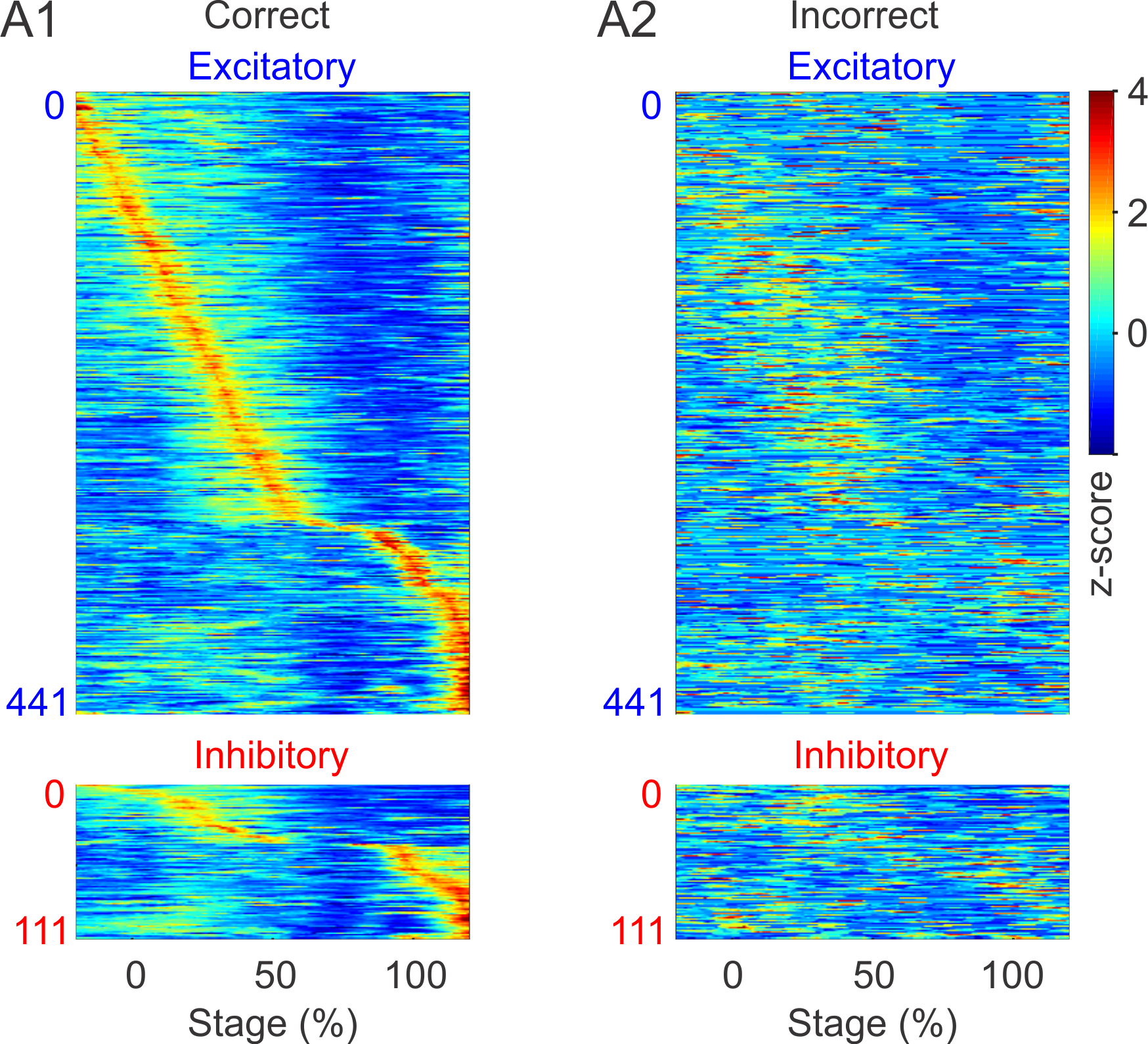

Supplement: Supplementary Figure S1 — PFC spiking activity during correct vs. incorrect trials. (Related to Figure 2). Normalized population firing rates of excitatory (blue) and inhibitory (red) neurons during correct trials (A1), and incorrect trials (A2). [file Image_1.JPEG]
